# The Cholesterol Transport Inhibitor U18666A Interferes with Pseudorabies Virus Infection

**DOI:** 10.3390/v14071539

**Published:** 2022-07-14

**Authors:** Byeongwoon Song

**Affiliations:** 1 Department of Microbiology, Immunology, and Physiology, Meharry Medical College, Nashville, TN 37208, USA; bsong@mmc.edu; Tel.: +1-(615)-327-6698; Fax: +1-(615)-327-6021; 2 Center for AIDS Health Disparities Research, Meharry Medical College, Nashville, TN 37208, USA

**Keywords:** alphaherpesvirus, pseudorabies virus, PRV, U18666A, cholesterol transport, lysosome

## Abstract

Many viruses require the maintenance of lysosomal cholesterol homeostasis for a successful infection; however, the role of lysosomal cholesterol homeostasis in the alphaherpesvirus life cycle is not clear. Here we show that the lysosomal cholesterol transport inhibitor U18666A interferes with the replication of pseudorabies virus (PRV), a member of the alphaherpesvirus subfamily. The treatment with U18666A caused a significant reduction in the production of infectious virus particles. The U18666A treatment was shown to suppress the release of PRV particles. Pretreating PRV virions with U18666A did not affect virus production, whereas pretreating target cells with U18666A led to a substantial reduction in virus yield. Our previous study showed that two cyclodextrin derivatives, 2-hydroxypropyl-β-cyclodextrin (HPβCD) and 2-hydroxypropyl-γ-cyclodextrin (HPγCD), can rescue the cholesterol accumulation defect in primary fibroblasts derived from a Niemann–Pick disease type C (NPC) patient. Here, we demonstrate that treatment with HPβCD or HPγCD not only rescues the U18666A-induced cholesterol accumulation but also rescues the U18666A-induced inhibition of PRV production. Collectively, our data suggest that U18666A interferes with PRV infection via altering cellular functions that are critical for the viral life cycle and may include lysosomal cholesterol homeostasis.

## 1. Introduction

The alphaherpesvirus subfamily includes herpes simplex virus type 1 and type 2 (HSV-1 and -2), varicella-zoster virus (VZV), and pseudorabies virus (PRV). Infections with the prototype alphaherpesvirus HSV-1 are very common, with a prevalence of 50–90% worldwide [1]. Primary infection with HSV-1 leads to life-long, persistent, latent infection, which can periodically reactivate [2]. Although the symptoms of HSV-1 infection commonly manifest as cold sores, it can be very severe in some cases, resulting in corneal blindness, lymphocytic meningitis, viral encephalitis, and even death [2]. Currently, there is no clinically approved vaccine for preventing HSV-1 infection. Nucleoside analogues, such as acyclovir and valacyclovir, are effective for treating the symptoms caused by HSV-1 infection [3]. However, severe side effects and drug resistance emerge after long-term use of these compounds and there has been a sharp increase in the onset of HSV resistance in immunocompromised patients [4]. There is a compelling need for the development of safer and more efficacious therapies for alphaherpesviruses, and agents with novel mechanisms of action are especially promising.

PRV is the causative agent of Aujeszky’s disease in pigs, which is characterized by encephalomyelitis, frequently accompanied by inflammation of the upper respiratory tract and lungs [5]. PRV infection also causes abortion and neonatal death and thus results in significant economic losses for the swine industry. PRV is an ideal tool to study basic mechanisms of alphaherpesvirus biology and has the enormous advantage of being an experimentally accessible natural virus–host system by infection of pigs. Moreover, its broad host range allows the use of other well-defined animal models, such as mouse and rat models, for neuroanatomical, immunological and molecular biological studies. Like other alphaherpesviruses, PRV exhibits a distinct neurotropism, invading the CNS via peripheral nerves, and has the ability to travel trans-synaptically, providing a useful tool in elucidating detailed neuroanatomical networks by using reporter-tagged viral vectors in mice and rats [6,7].

During herpesvirus infection, infectious particles attach to the cell receptor and the viral envelope fuses with the cell membrane and the capsid and tegument enter the cell [8]. Entry by endocytosis also occurs in some cell types. Nucleocapsids are released into the cytosol and trafficked to the nucleus, which is followed by entry of the viral DNA into the nucleus, transcription of viral genes, replication of viral DNA, and nucleocapsid assembly. Nucleocapsids egress from the nuclear membranes after envelopment and de-envelopment. Eventually, the tegument layer covers nucleocapsids in the cytosol, and virions become mature by secondary envelopment in the trans-Golgi network [8].

Membrane cholesterol is a crucial molecule affecting interactions of microbial pathogens with mammalian cells [9]. Cholesterol trafficking and distribution are vital for many cellular functions [10,11]. Lysosomes play a critical role in the maintenance of intracellular cholesterol homeostasis in addition to their canonical functions such as the turnover of macromolecules, membrane repair, nutrient sensing, and cell death [12,13]. Mammalian cells acquire cholesterol from endocytosed low-density lipoproteins (LDL). Following entry into the cell via LDL receptor-mediated endocytosis, the LDL-associated cholesteryl ester is transported into early endosomes, late endosomes and lysosomes, and unesterified free cholesterol is then exported from the late endosomes and lysosomes via the concerted action of the lysosomal proteins NPC1 [14] and NPC2 [15]. A critical role of lysosomal cholesterol homeostasis in virus replication has been demonstrated for several viruses. For example, NPC1 deficiency interferes with the release of human immunodeficiency virus type 1 (HIV-1) [16]. Hepatitis C virus (HCV) replication depends on the proteins involved in endosomal/lysosomal cholesterol trafficking including NPC1 [17]. Furthermore, several drugs under testing for the therapy of severe acute respiratory syndrome coronavirus 2 (SARS-CoV-2), the etiological agent of the COVID-19 pandemic, disrupt the endosomal/lysosomal cholesterol homeostasis [18,19,20]. However, the functional significance of lysosomal cholesterol homeostasis for the replication cycle of alphaherpesviruses remains to be determined. In the present study, we explored the impact of disrupting lysosomal cholesterol trafficking on PRV infection. Here we show that lysosomal cholesterol homeostasis plays an important role in the PRV replication cycle.

## 2. Results

### 2.1. U18666A Causes Accumulation of Intracellular Cholesterol

**Figure 1:**
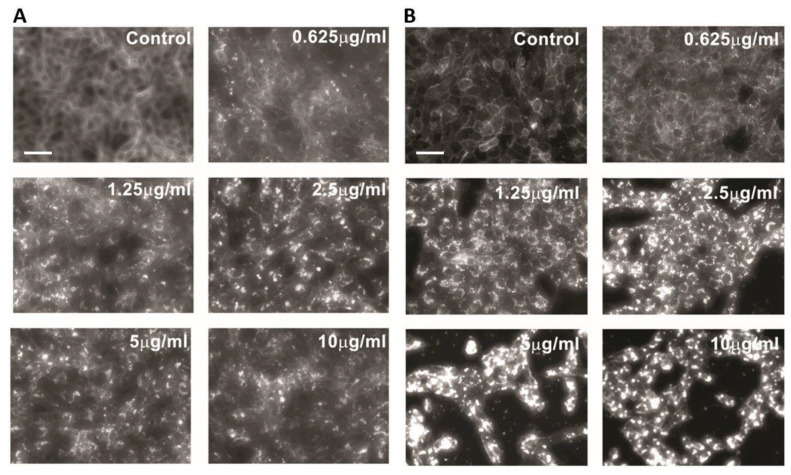
U18666A causes accumulation of intracellular cholesterol. PK-15 (**A**) or Vero (**B**) cells were treated with U18666A (0.625–10 μg/mL) or DMSO (0.1%, *v*/*v*) as a vehicle control for 48 h. Unesterified (free) cholesterol was detected by staining with filipin. Data are representative of three independent experiments. Scale bar = 50 µm.

To investigate the impact of lysosomal cholesterol accumulation on PRV replication, we used the cationic amphiphilic compound U18666A, an inhibitor of lysosomal cholesterol export [21,22]. Two different cell lines that support PRV replication, the porcine kidney epithelial cell line PK-15 and the African green monkey kidney epithelial cell line Vero, were treated with increasing concentrations of U18666A for 48 h and subjected to staining for unesterified, free cholesterol by using filipin (Figure 1). As expected, U18666A treatment caused the accumulation of intracellular cholesterol in both PK-15 (Figure 1A) and Vero (Figure 1B) cells. In contrast, cells treated with DMSO (0.1%, *v*/*v*) as a vehicle control showed filipin stain localized on the plasma membrane or diffused throughout the cytoplasm without intracellular cholesterol accumulation. These results are consistent with the role of U18666A in blocking the egress of cholesterol from lysosomes via inhibiting the cholesterol transporter NPC1 [21].

### 2.2. U18666A Inhibits Production of Infectious PRV Particles

**Figure 2:**
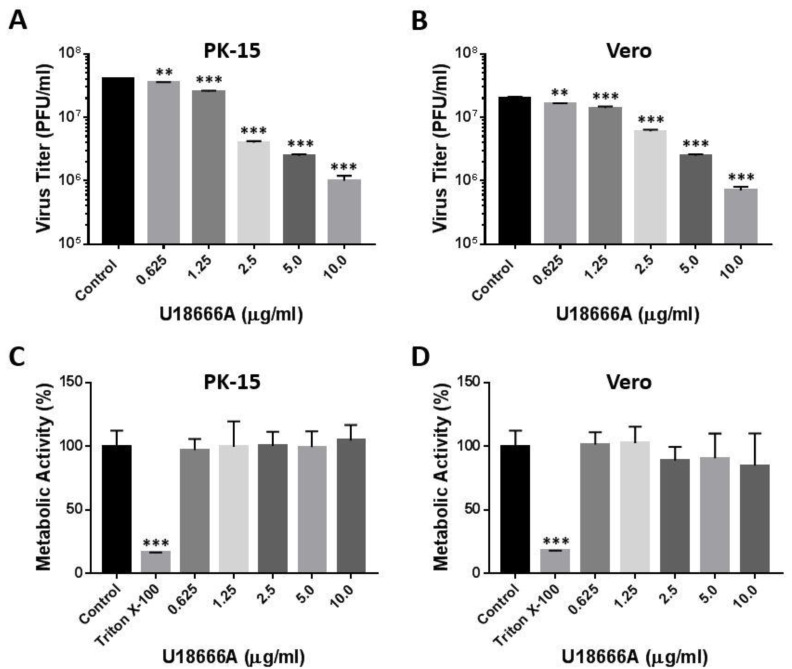
U18666A inhibits production of infectious PRV particles. PK-15 (**A**) or Vero (**B**) cells were infected with PRV at an MOI of 5 in the presence of U18666A (0.625–10 μg/mL) or DMSO (0.1%, *v*/*v*) as a control. At 48 h post-infection, virus titer in the culture supernatant was measured by plaque assay. Virus titer is presented in plaque-forming units (PFU)/mL. Data are mean ± SD (*n* = 3). ** *p* < 0.01; *** *p* < 0.001. Metabolic activity of PK-15 (**C**) or Vero (**D**) cells was measured 48 h after U18666A treatment using the Cell Titer 96 Aqueous One Solution Cell Proliferation Assay (Promega). DMSO (0.1%, *v*/*v*) and Triton X-100 (0.1%, *v*/*v*) were included as negative and positive controls for cell cytotoxicity. The level of metabolic activity was normalized to the DMSO-treated control (100%). Data are mean ± SD (*n* = 3). *** *p* < 0.001.

We next tested whether U18666A interferes with the replication cycle of PRV. PK-15 and Vero cells were pretreated with increasing concentrations of U18666A (0.625–10 μg/mL) for 2 h and infected with PRV (Becker strain) at a multiplicity of infection (MOI) of 5 for 48 h in the presence of the compound. The levels of infectious progeny virions released into the culture supernatant were determined by plaque assay. U18666A treatment reduced the virus titer in both PK-15 cells (Figure 2A) and Vero cells (Figure 2B) in a dose-dependent manner. To exclude the possibility of unintended cell toxicity by U18666A treatment, an MTS-based cell viability assay was conducted. The treatment of cells with U18666A did not affect the cell viability, as assessed by metabolic activity, at the concentrations used in this study in both PK-15 (Figure 2C) and Vero cells (Figure 2D). As expected, Triton X-100 (0.1%; *v*/*v*), used as a positive control for cytotoxicity, significantly reduced the cell viability in both cell types. These results indicate that U18666A inhibits PRV infection without causing cell toxicity.

**Figure 3:**
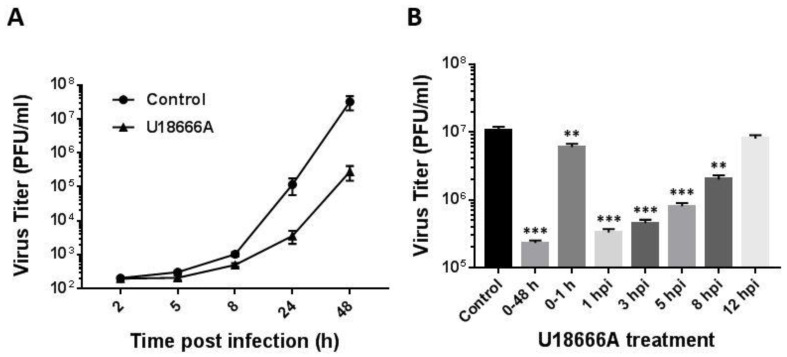
Effects of U18666A on PRV replication. (**A**) PK-15 cells were infected with PRV at an MOI of 5 in the presence of U18666A (5 μg/mL) or DMSO (0.1%, *v*/*v*) as a control. At the indicated times post-infection, virus titer in the culture supernatant was measured by plaque assay. Virus titer is presented in plaque-forming units (PFU)/mL. Data are mean ± SD (*n* = 3). (**B**) PK-15 cells were infected with PRV at an MOI of 5 and U18666A (5 μg/mL) was added at the indicated times during or after infection. Treatment with DMSO (0.1%, *v*/*v*) was included as a control. Virus titer in the culture supernatant was determined by plaque assay at 48 h post-infection. Data are mean ± SD (*n* = 3). ** *p* < 0.01; *** *p* < 0.001.

We next determined the impact of U18666A on the replication kinetics of PRV in PK-15 cells by measuring the virus yield at 2, 5, 8, 24 and 48 h post-infection (Figure 3A). While the release of infectious PRV particles started to increase at 8 h post-infection in both the DMSO-treated control and U18666A-treated group, the virus yield of U18666A-treated cells lagged behind that of the control group at later time points.

To examine the time period in which U18666A exerts antiviral activity, we conducted a time-of-addition experiment with U18666A (Figure 3B). PK-15 cells were inoculated with PRV at an MOI of 5 for 1 h in the absence or presence of U18666A (5 μg/mL). After washing with phosphate-buffered saline, the infected cells were cultured in a fresh medium with U18666A (5 μg/mL) added at the indicated times post-infection. At 48 h post-infection, virus yield in the culture supernatant was determined by plaque assay. When U18666A was present throughout the entire infection period (0–48 h), virus titer was reduced substantially compared with the DMSO-treated control. The antiviral potency was gradually reduced in a time-dependent manner when U18666A was added at later time points after infection. While the treatment of cells with U18666A at 1 h post-infection (1 hpi) showed inhibition of viral replication similar to that of the cells treated with the drug for the entire culture period (0–48 h), the treatment of cells with U18666A during the first one hour only (0–1 h) caused a modest reduction in virus yield compared to the DMSO-treated control. These findings suggest that the disruption of lysosomal cholesterol transport may interfere with PRV infection at multiple stages of the virus life cycle including the early and late steps.

### 2.3. Maintenance of Lysosomal Cholesterol Homeostasis Is Important for Productive Replication of PRV

**Figure 4:**
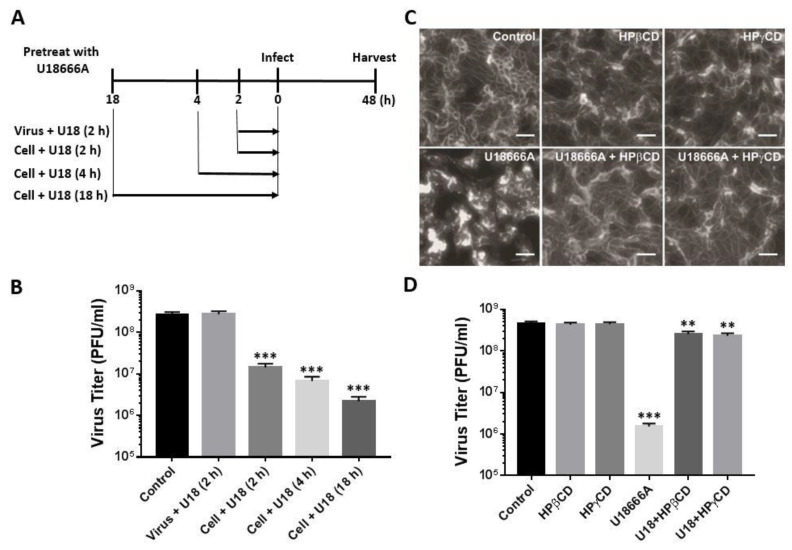
Lysosomal cholesterol homeostasis is critical for PRV infection. (**A**) A schematic diagram for testing the impact of pretreating virus or the target cells with U18666A (5 μg/mL) on PRV infection. (**B**) Infection of PK-15 cells with PRV (5 MOI) in the presence of U18666A (5 μg/mL) was conducted following the scheme outlined in (**A**). Virus titer in the culture supernatant was determined by plaque assay at 48 h post-infection. Treatment with DMSO (0.1%, *v*/*v*) was included as a control. U18: U18666A. Data are mean ± SD (*n* = 3). *** *p* < 0.001. (**C**) PK-15 cells were treated with HPβCD (1 mM), HPγCD (1 mM), U18666A (5 μg/mL) or U18666A in combination with HPβCD or HPγCD for 48 h. Treatment with DMSO (0.1%, *v*/*v*) was included as a control. Unesterified (free) cholesterol was detected by staining with filipin. Scale bar = 50 µm. (**D**) PK-15 cells were treated with HPβCD (1 mM), HPγCD (1 mM), U18666A (5 μg/mL) or U18666A in combination with HPβCD or HPγCD for 2 h, and then the cells were infected with PRV (MOI = 5) in the presence of the drugs. At 48 h post-infection, virus titer in the culture supernatant was determined by plaque assay. Treatment with DMSO (0.1%, *v*/*v*) was included as a control. U18: U18666A. Data are mean ± SD (*n* = 3). ** *p* < 0.01; *** *p* < 0.001.

We tested whether U18666A inhibits PRV (Becker) infection by abrogating the virion structure/infectivity or by altering cellular functions that are critical to PRV infection. To this end, we pretreated PRV for 2 h or pretreated the target cell PK-15 for 2, 4 or 18 h with U18666A prior to infection. At 48 h post-infection, virus yield in the culture supernatant was determined by plaque assay. Pretreating virions with U18666A did not affect the virus yield, whereas pretreating the target cells with U18666A inhibited PRV infection in a time-dependent manner (Figure 4A,B). These results suggest that U18666A exerts an antiviral effect through altering cellular pathways or functions that are critical for PRV infection, not through abrogating the infectivity or structural integrity of PRV particles.

NPC1 is a lysosomal membrane protein that is involved in the exit of LDL-derived cholesterol from lysosomes [13]. We recently demonstrated that two cyclodextrin derivatives, 2-hydroxypropyl-β-cyclodextrin (HPβCD) and 2-hydroxypropyl-γ-cyclodextrin (HPγCD), can rescue the cholesterol accumulation defect in primary fibroblasts derived from NPC1 patient [23,24]. We, therefore, determined whether HPβCD and HPγCD are able to rescue the U18666A-induced cholesterol accumulation in PK-15 cells (Figure 4C). Treatment with HPβCD or HPγCD alone did not affect the cholesterol distribution pattern compared with the DMSO-treated control (Figure 4C, top). As expected, the treatment with U18666A induced intracellular cholesterol accumulation and the co-treatment with HPβCD or HPγCD rescued the U18666A-induced cholesterol accumulation (Figure 4C, bottom). We next determined whether HPβCD or HPγCD treatment can rescue the U18666A-induced inhibition of PRV infection (Figure 4D). PK-15 cells were pretreated with U18666A, HPβCD, HPγCD or U18666A in combination with HPβCD or HPγCD for 2 h and infected with PRV at an MOI of 5 in the presence of the drugs. The production of infectious progeny virions in the culture supernatant was determined at 48 h post-infection by plaque assay. The treatment with U18666A significantly reduced the virus yield, whereas treatment with HPβCD or HPγCD did not affect the virus yield. Interestingly, co-treatment with HPβCD or HPγCD in combination with U18666A relieved the U18666A-induced inhibition of PRV infection. These results underscore the importance of lysosomal cholesterol homeostasis for the productive replication of PRV.

### 2.4. Effects of U18666A on PRV 1028 Infection

We tested the impact of U18666A on the production of infectious virus particles using a recombinant PRV 1028 that expresses a carboxy-terminal VP26-mCherry fusion [25]. Compared to previously characterized recombinants expressing N-terminal fusions, PRV 1028 expresses more VP26 fusion protein and incorporates more VP26 fusion protein into virus particles, and individual virus particles exhibit brighter red fluorescence.

**Figure 5:**
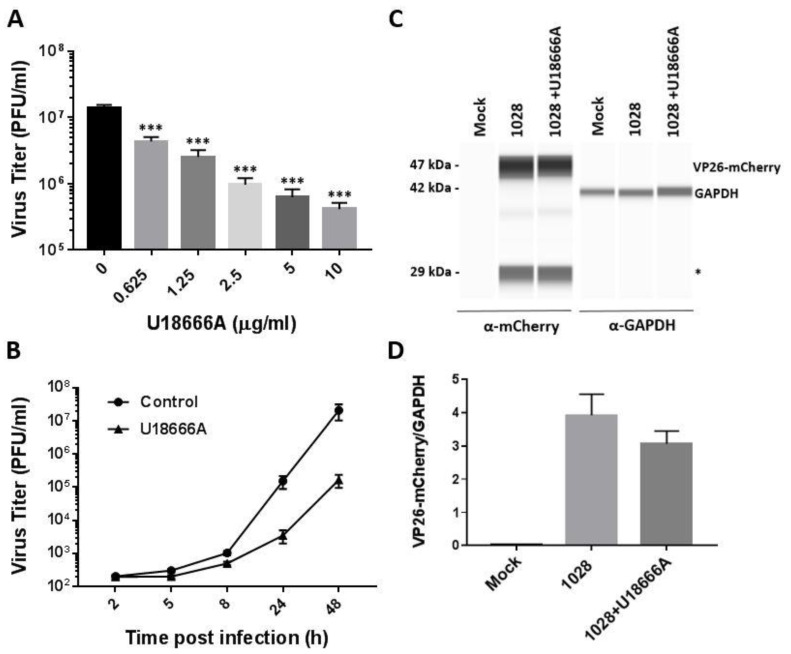
Effects of U18666A on PRV1028 production and protein expression. (**A**) PK-15 cells were infected with PRV1028, a recombinant PRV expressing a carboxy-terminal VP26-mCherry fusion, at an MOI of 5 in the presence of U18666A (0.625–10 μg/mL) or DMSO (0.1%, *v*/*v*) as a control. At 48 h post-infection, virus titer in the culture supernatant was measured by plaque assay. Virus titer is presented in plaque-forming units (PFU)/mL. Data are mean ± SD (*n* = 3). *** *p* < 0.001. (**B**) PK-15 cells were infected with PRV 1028 at an MOI of 5 in the presence of U18666A (5 μg/mL) or DMSO (0.1%, *v*/*v*) as a control. At the indicated times post-infection, virus titer in the culture supernatant was measured by plaque assay. Data are mean ± SD (*n* = 3). (**C**) Effects of U18666A on the PRV 1028 VP26-mCherry protein expression. PK-15 cells were infected with PRV 1028 (MOI = 5) in the presence of U18666A (5 μg/mL) or DMSO (0.1%, *v*/*v*) as a control. Cell extracts were harvested at 24 h post-infection and subjected to WES protein analysis (ProteinSimple, San Jose, CA, USA) using rabbit anti-mCherry antibody (Abcam ab183628). Probing with anti-GAPDH antibody (Cell Signaling #5174) was included as a loading control. A mock-infected sample was included as a negative control. The asterisk (*) indicates the position of the degradation product from VP26-mCherry. A full image of WES protein analysis can be found in Supplementary Materials. Data are representative of three independent experiments. (**D**) The intensity of protein bands for VP26-mCherry and GAPDH was quantitated using the WES protein analysis software (ProteinSimple), and the ratio of VP26-mCherry/GAPDH was determined. Data are mean ± SD (*n* = 3) from three replicate runs in a representative experiment.

PK-15 cells were pretreated with increasing concentrations of U18666A (0.625–10 μg/mL) for 2 h and infected with PRV 1028 at an MOI of 5 for 48 h in the presence of the compound. The titer of infectious progeny virions released into the culture supernatant was determined by plaque assay. The treatment with U18666A showed a dose-dependent inhibition of virus production (Figure 5A). We next examined viral replication kinetics in the presence of U18666A. While the release of infectious virus particles started to increase at 8 h post-infection in both the DMSO-treated control and U18666A-treated group, the virus yield of U18666A-treated cells lagged behind that of the control group at later time points (Figure 5B).

We next determined the level of VP26-mCherry protein expression in PK-15 cells infected with PRV 1028 in the absence or presence of U18666A (5 μg/mL). The treatment with U18666A caused a modest reduction in the level of VP26-mCherry at 24 h post-infection compared with the DMSO-treated control (Figure 5C,D).

**Figure 6:**
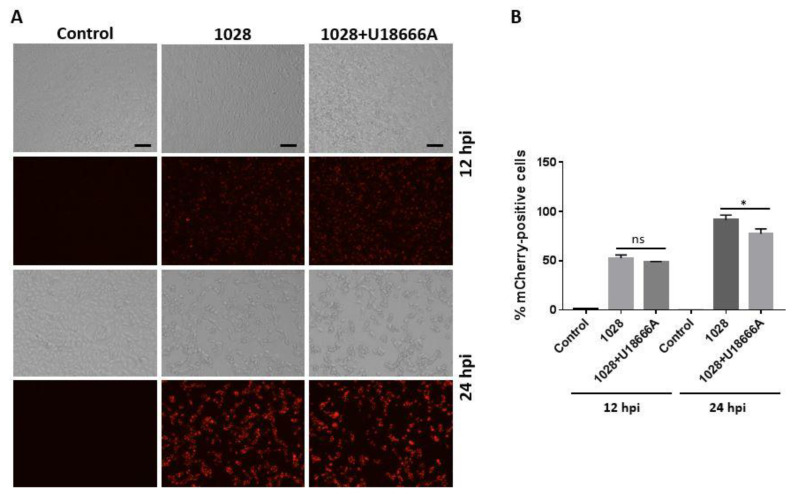
U18666A treatment causes a reduction in VP26-mCherry signal in PK-15 cells infected with PRV 1028. (**A**) PK-15 cells were infected with PRV 1028 (MOI = 5) in the presence of U18666A (5 μg/mL) or DMSO (0.1%, *v*/*v*) as a control. Infected cells were subjected to fluorescent microscopy using the EVOS cell imaging system (Thermo Fisher Scientific, Waltham, MA, USA). Mock infection was included as a negative control. Images were obtained at 12 h and 24 h post-infection. Data are representative of three independent experiments. Scale bar = 100 µm. (**B**) PK-15 cells were infected with PRV 1028 (MOI = 5) in the presence of U18666A (5 μg/mL) or DMSO (0.1%, *v*/*v*) as a control. At 12 and 24 h post-infection, cells were harvested and analyzed using CellStream Flow Cytometer (Luminex, Austin, TX, USA) according to the manufacturer’s instructions and the percentage of mCherry-positive cells was determined with excitation wavelength at 587 nm and emission wavelength at 610 nm. Data are mean ± SD (*n* = 3). * *p* < 0.05.

We further tested the impact of U18666A on PRV 1028 infection by monitoring the red fluorescent protein mCherry. PK-15 cells were infected with PRV 1028 at an MOI of 5 in the presence of U18666A (5 μg/mL) or DMSO (0.1%, *v*/*v*) as a control and the infected cells were subjected to fluorescence microscopy (Figure 6A) and flow cytometry (Figure 6B). At 12 h post-infection, the number of cells expressing the VP26-mCherry protein seemed to be similar whether the infection was conducted in the absence or presence of U18666A (Figure 6A, top). At 24 h post-infection, the number of cells expressing the VP26-mCherry protein was modestly reduced by the U18666A treatment (Figure 6A, bottom). As expected, a red fluorescence signal was absent in the mock infection control. The measurement of the percentage of mCherry-positive cells by flow cytometer showed that the treatment with U18666A causes a modest reduction in the number of mCherry-positive cells at 24 h post-infection while there was no difference at 12 h post-infection (Figure 6B).

### 2.5. Effects of U18666A on the Assembly and Release of PRV

**Figure 7:**
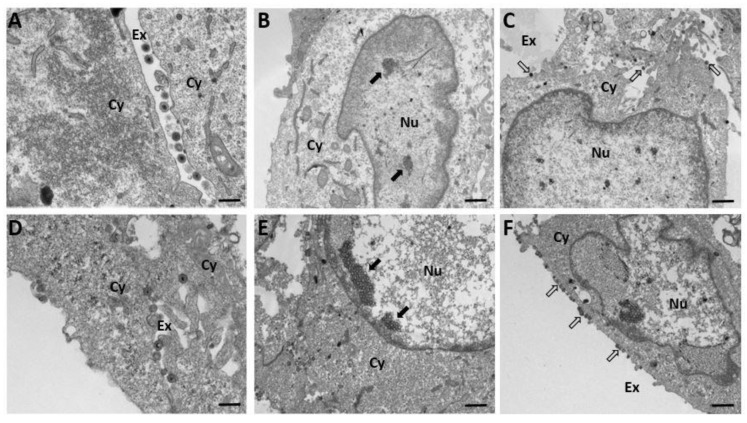
Transmission electron microscopy on PRV assembly in PK-15 cells. PK-15 cells were infected with PRV (Becker) at an MOI of 5 in the presence of U18666A (5 μg/mL) or DMSO (0.1%, *v*/*v*) control. Samples were harvested at 12 h post-infection and examined under transmission electron microscopy as described in Materials and Methods. The images obtained at higher magnification (**A**,**D**) show that mature PRV particles of similar size and morphology are produced from the DMSO-treated control (**A**) and the U18666A-treated group (**D**). For the images obtained at lower magnification (**B**,**C**,**E**,**F**), the black arrows indicate a cluster of PRV capsids in the nucleus of the DMSO-treated control (**B**) and the U18666A-treated group (**E**) while the white arrows indicate PRV particles in the extracellular space or on the plasma membrane of the DMSO-treated control (**C**) and the U18666A-treated group (**F**). Nu, nucleus; Cy, cytoplasm; Ex, extracellular space. Scale bar in (**A**,**D**), 400 nm. Scale bar in (**B**,**C**,**E**, **F**), 1 μm.

To investigate whether the disruption of lysosomal cholesterol transport affects the assembly and the morphology of PRV, intracellular and extracellular virus particles were examined using transmission electron microscopy. PK-15 cells were infected with PRV (Becker) at an MOI of 5 in the presence of U18666A (5 μg/mL) or DMSO (0.1%, *v*/*v*) as a control. Samples were harvested at 12 h post-infection and examined under transmission electron microscopy as described in Materials and Methods. Both the DMSO-treated control (Figure 7A) and the U18666A-treated group (Figure 7D) produced mature virus particles released into the extracellular space; the size and morphology of the virus particles produced from the U18666A-treated group were similar to those produced from the DMSO-treated control. A cluster of viral capsids was observed in the nucleus of both the DMSO-treated control (Figure 7B,C) and the U18666A-treated group (Figure 7E,F). It is of interest to observe that a much larger cluster of viral capsids was observed within the nucleus in the U18666A-treated group (Figure 7E, black arrow) compared with the DMSO-treated control (Figure 7B) and that many virus particles were tethered to the plasma membrane in the U18666A-treated group (Figure 7F, white arrow) compared with the DMSO-treated control (Figure 7C). These results raise the possibility that lysosomal cholesterol homeostasis may be involved in the assembly and/or release of PRV particles.

**Figure 8:**
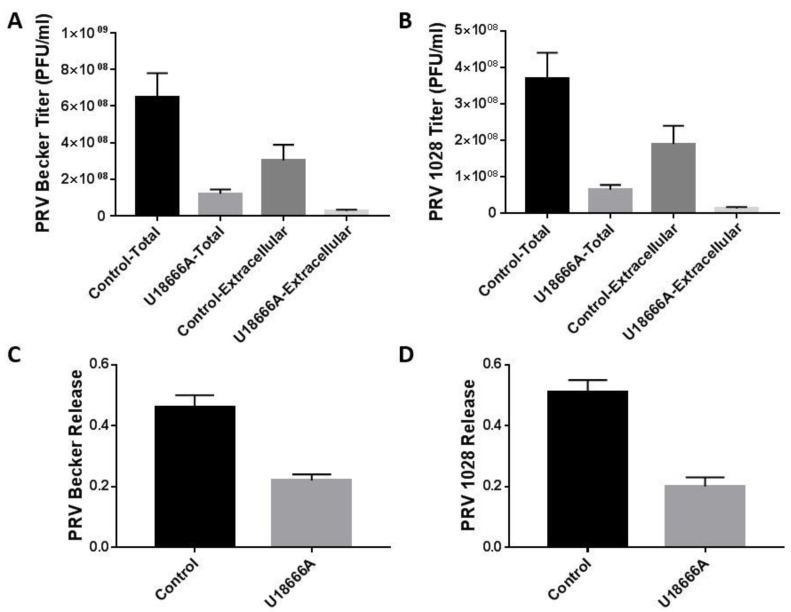
U18666A treatment suppresses the release of infectious PRV particles. PK-15 cells were infected with PRV at an MOI of 5 in the presence of U18666A (5 μg/mL) or DMSO (0.1%, *v/v*) as a control. Samples (cells and supernatants) were harvested at 24 h post-infection. (**A**,**B**) The levels of infectious virus particles in the cell and supernatant (extracellular) fractions were determined by plaque assay. The total virus titer was defined as the combined virus titer from the cell and the extracellular fractions. (**C**,**D**) Virus release was defined as the ratio of the virus titer in the extracellular fraction and the virus titer in the total fraction: Release = [virus titer in extracellular fraction]/[virus titer in total fraction]. Data are mean ± SD (*n* = 3).

We determined whether U18666A treatment interferes with the release of PRV particles. PK-15 cells were infected with PRV Becker or 1028 in the presence of U18666A or DMSO and the virus yield in the total (cell and extracellular) and the extracellular fractions was determined at 24 h post-infection. As expected, the U18666A treatment caused a substantial reduction of virus titer in the extracellular and total fractions for both PRV Becker (Figure 8A) and PRV 1028 infection (Figure 8B). Virus release was determined by dividing the virus yield in the extracellular fraction by the virus yield in the total fraction. While approximately 50% of infectious virus particles were released into the culture supernatant in the DMSO-treated control, only 20% of infectious virus particles were released into the culture supernatant in the U18666A-treated group for both PRV Becker (Figure 8C) and PRV 1028 infection (Figure 8D). These data strongly suggest that lysosomal cholesterol homeostasis is important for the efficient release of PRV particles.

## 3. Discussion

Cholesterol is an important component of the cellular and viral membranes. Cholesterol-containing intracellular compartments also play a critical role in the replication of several viruses. Alphaherpesviruses require cell membrane cholesterol for entry into target cells [26,27,28,29,30,31]. For example, the entry of HSV-1 and PRV requires target membrane cholesterol at the fusion step [26,28]. During cell entry, alphaherpesviruses use low-pH endosomal pathways or pH-independent routes, both of which require cholesterol [32,33,34,35]. It is known that alphaherpesviruses acquire their envelopes from the internal membranes of infected cells during the assembly of new progeny virus [36,37]. Recent studies suggested that cholesterol can influence steps after incoming capsid arrival at the nucleus, including viral protein expression, the release of mature progeny virus and cell-to-cell spread [38]. The cationic amphiphilic compound U18666A is known to block the exit of cholesterol from lysosomes via inhibiting the cholesterol transporter NPC1 [21]. Here we explored the impact of disrupting lysosomal cholesterol trafficking on PRV infection using U18666A. Our study provides evidence that lysosomal cholesterol homeostasis is important for the productive replication of PRV.

When cells were infected with PRV in the presence of the lysosomal cholesterol transport inhibitor U18666A, there was a substantial reduction in the production of infectious PRV particles measured by plaque assay. The WES protein analysis as well as fluorescent microscopy and flow cytometry study indicated that U18666A causes a modest reduction in the PRV small capsid protein expression. The virus release assay showed that the release of infectious PRV particles is suppressed by the U18666A treatment. A transmission electron microscopy study suggested that virus assembly and/or release may be affected in the infected cells treated with U18666A. It remains to be determined whether the clustering of viral capsids within the nucleus or the tethering of virus particles to the plasma membrane, observed in transmission electron microscopy study, is directly linked to the U18666A-induced suppression of production of infectious virus particles. HPβCD and HPγCD rescue of U18666A-induced cholesterol accumulation and viral inhibition strongly suggested that lysosomal cholesterol homeostasis is important for the productive replication of PRV.

A recent study by Li et al. demonstrated that U18666A is highly potent in suppressing PRV and HSV-1 infection and that the antiviral activity of U18666A is dependent on NPC1 [39]. While Li et al.’s study agrees overall with our study, there are minor discrepancies between the two studies. For example, our study indicated that U18666A suppresses PRV infection at multiple stages including virus release, whereas Li et al.’s work showed that U18666A blocks viral entry and does not affect viral replication or release. The differences in virus strains and experimental strategies used in each study may have contributed to the discrepancies. First, as an attempt to analyze the early steps of viral infection, an EdU-labeled PRV was used in Li et al.’s study, whereas a recombinant PRV expressing VP26-mCherry was used in our study. Second, we used a plaque assay to determine infectious virus titers whereas Li et al.’s work used a 50% tissue culture infective dose (TCID_50_) assay to assess virus titers. Despite the minor discrepancies, both our and Li et al.’s studies suggest that intracellular cholesterol homeostasis is critical for alphaherpesvirus infection.

The study presented here demonstrates that the lysosomal cholesterol transport inhibitor U18666A interferes with the replication cycle of PRV. The U18666A treatment seems to suppress the production of mature PRV particles by altering cellular functions that are important for the viral life cycle and may include lysosomal cholesterol trafficking. Our data suggest that lysosomal cholesterol homeostasis is critical for the efficient release of PRV particles. The blockage of cholesterol exit from lysosomes induced by U18666A could disrupt intracellular cholesterol distribution and protein/vesicle trafficking. These cellular alterations may in turn interfere with one or more steps of the viral life cycle that include virus assembly or release. Further work is warranted to define the viral and cellular determinants responsible for the inhibition of PRV infection by the U18666A treatment. Defining the molecular mechanisms by which U18666A-induced disruptions in intracellular cholesterol homeostasis influence PRV infection will enhance the understanding of virus–host cell interactions and contribute to the development of novel therapeutic approaches for alphaherpesviruses.

## 4. Materials and Methods

### 4.1. Chemicals and Reagents

Cell culture media and reagents were purchased from Thermo Fisher Scientific (Waltham, MA, USA). These include Dulbecco’s modified Eagle’s medium (DMEM), fetal bovine serum (FBS), and penicillin/streptomycin. A CellTiter 96 Aqueous One Solution Cell Proliferation Assay System was purchased from Promega (Madison, WI, USA). U18666A, Filipin III, 2-hydroxypropyl-β-cyclodextrin (HPβCD) and 2-hydroxypropyl-γ-cyclodextrin (HPγCD) were obtained from Sigma-Aldrich (St. Louis, MO, USA). Primary antibodies used in this study are as follows: rabbit polyclonal antibody for mCherry (Abcam ab183628); rabbit monoclonal antibody for GAPDH (Cell Signaling #5174).

### 4.2. Cells and Viruses

Pig kidney cell line PK-15 was provided by Dr Lynn W. Enquist (Princeton University). African green monkey kidney cell line Vero (CRL-1586) was obtained from the American Type Culture Collection (ATCC). Cells were cultured in DMEM containing 10% FBS, 100 U/mL of penicillin and 100 μg/mL of streptomycin, and maintained at 37 °C in a 5% CO_2_ humidified incubator.

PRV strains Becker and 1028 were provided by Dr Lynn W. Enquist (Princeton University). PRV1028 is a recombinant virus expressing a carboxy-terminal VP26-mCherry fusion [25]. PRV Becker and 1028 were propagated in PK-15 cells. Viral titers of PRV were determined by plaque assay in Vero cells. Briefly, the confluent Vero cells were infected with serial dilutions of PRV and overlaid with DMEM containing 2% FBS and 1% agarose. After 48–72 h incubation, the cells were fixed with 4% formaldehyde in PBS and stained with 0.5% crystal violet in 10% methanol. After rinsing with water and air drying, the plaques were counted.

### 4.3. Cell Viability Assay

Cells were seeded overnight in 96-well plates at a density of 2–3 × 10^4^ cells per well. Cells were treated with a series of dilutions of the test compounds. Cells treated with DMSO (0.1%, *v*/*v*) and cells treated with Triton X-100 (0.1%, *v*/*v*) were included as negative and positive controls, respectively. After 24–48 h incubation at 37 °C, cell viability was determined using the CellTiter 96 Aqueous One Solution Cell Proliferation Assay (Promega), which is based on the conversion of the tetrazolium compound [3-(4,5-dimethylthiazol-2-yl)-5-(3-carboxymethoxyphenyl)-2-(4-sulfophenyl)-2H-tetrazolium, inner salt; MTS], as per the manufacturer’s instructions. The formation of colored formazan product by dehydrogenase enzymes in metabolically active cells was recorded as absorbance at 490 nm using Synergy HT microplate reader (BioTek, Winooski, VT, USA). The data were normalized to the values for the DMSO-treated control.

### 4.4. Intracellular Accumulation of Unesterified Cholesterol

Cells cultured in glass-bottom 24-well plates were fixed as previously described [24]. Cells were stained with Filipin III (12.5 μg/mL in PBS) for 45 min at room temperature. After washing three times with PBS, cells were mounted in anti-fade mounting medium. Images were acquired using a Nikon TE2000 wide-field microscope with standard filter sets using 20× objective and analyzed using Nikon image software.

To test the ability of HPβCD and HPγCD to rescue U18666A-induced cholesterol accumulation, PK-15 cells were treated with HPβCD (1 mM), HPγCD (1 mM), U18666A (5 μg/mL) or U18666A in combination with HPβCD or HPγCD for 48 h. The cells were then subjected to filipin staining and microscopy to detect cholesterol accumulation.

### 4.5. Antiviral Assay

PK-15 or Vero cells were infected with PRV at a multiplicity of infection (MOI) of 5 in the presence of U18666A at the indicated concentrations or DMSO (0.1%, *v*/*v*) as a vehicle control. At 48 h post-infection, virus yield in the culture supernatant was determined by plaque assay. We determined the impact of U18666A on replication kinetics of PRV in PK-15 cells by measuring the virus yield in the culture supernatant at 2, 5, 8, 24 and 48 h post-infection.

To identify the stages of the PRV replication cycle affected by U18666A, PK-15 cells were infected with PRV at an MOI of 5 in the absence or presence of U18666A for 1 h and washed with PBS, and then U18666A was added at different time points after infection. To test the impact of U18666A on virions, 100-fold concentrated virions were incubated with U18666A for 2 h at 37 °C prior to infection. The U18666A-treated, concentrated virions were 100-fold diluted prior to infecting the target cells at an MOI of 5. To test the impacts of U18666A on the target cells, PK-15 cells were incubated with U18666A for 2, 4 or 18 h at 37 °C prior to infection. The cells were washed with PBS and infected with PRV at an MOI of 5.

To test the ability of HPβCD and HPγCD to rescue the U18666A-induced inhibition of PRV infection, PK-15 cells were treated with HPβCD (1 mM), HPγCD (1 mM), U18666A (5 μg/mL) or U18666A in combination with HPβCD or HPγCD for 2 h, and then the cells were infected with PRV at an MOI of 5 in the presence of the drugs. At 48 h post-infection, virus yield in the culture supernatant was determined by plaque assay.

### 4.6. Protein Analysis

The protein levels in cells were determined by WES protein analysis (ProteinSimple, San Jose, CA, USA) according to the manufacturer’s instructions. Briefly, cells were washed with ice-cold PBS and lysed in MPER lysis buffer (Thermo Fischer Scientific) containing protease inhibitors cocktail (Thermo Fischer Scientific) by incubating for 15 min on ice. The cell debris was removed by centrifugation at 10,000× *g* for 15 min at 4 °C. The protein levels in clear cell lysate were measured by Bradford assay (VWR Life Science). Total protein (250 or 500 ng) was separated and immunoprobed in a capillary system using the WES ProteinSimple system: rabbit polyclonal antibody for mCherry (Abcam ab183628) and rabbit monoclonal antibody for GAPDH (Cell Signaling #5174) were used at 1:50 or 1:100 dilutions. Quantitative results such as molecular weight and signal intensity (area) were obtained using the WES ProteinSimple system software according to the manufacturer’s instructions.

### 4.7. Fluorescence Microscopy

PK-15 cells were infected with PRV1028, a recombinant PRV expressing a carboxy-terminal VP26-mCherry fusion [25], at an MOI of 5 in the presence of U18666A (5 μg/mL) or DMSO (0.1%, *v*/*v*). Red fluorescence and phase-contrast images were obtained at 12 h and 24 h post-infection using EVOS fl cell imaging system (Thermo Fisher Scientific).

### 4.8. Flow Cytometry

PK-15 cells were infected with PRV1028 at an MOI of 5 in the presence of U18666A (5 μg/mL) or DMSO (0.1%, *v*/*v*). At 12 and 24 h post-infection, the cells were analyzed by CellStream Flow Cytometer (Luminex, Austin, TX, USA). Initial gating on cells was set using FSC and SSC parameters. The percentage of mCherry-positive cells was analyzed using zone D and channel 5 of the CellStream with excitation wavelength at 587 nm and emission wavelength at 610 nm.

### 4.9. Transmission Electron Microscopy

All EM reagents were purchased from Electron Microscopy Sciences (Hatfield, PA, USA). The cells were fixed in 2.5% glutaraldehyde in 0.1 M cacodylate for 1 h. Samples were postfixed with 1% tannic acid in cacodylate for 1 h and then 1% uranyl acetate in ddH_2_O for 30 min, followed by dehydration in a graded ethanol series. After dehydration, the samples were transitioned to propylene oxide and infiltrated with a Quetol 651 formulated Spurr’s resin [40]. The resin was polymerized at 60 °C for 48 h. Thin sections were cut at a nominal thickness of 70 nm and poststained with 2% uranyl acetate and Reynold’s lead citrate. TEM imaging was performed using an FEI Technai T-12 transmission electron microscope operating at 100 kV using a side-mounted AMT CCD camera.

### 4.10. Statistical Analysis

Statistical analysis was conducted as previously described [24]. Results are expressed as mean ± standard deviation (S.D.) from 3 independent experiments (*n* = 3). For comparisons, the statistical significance of differences in mean values was determined by an unpaired t-test using GraphPad Prism 7 (GraphPad software, La Jolla, CA, USA). A *p*-value of 0.05 or less was considered statistically significant.

## Data Availability

Not applicable.

## Supplementary Materials

The following supporting information can be downloaded at: https://www.mdpi.com/article/10.3390/v14071539/s1, Figure S1: A full image of WES protein analysis.
