# Supplementary material for: The Cholesterol Transport Inhibitor U18666A Interferes with Pseudorabies Virus Infection
Source: Viruses. 2022 Jul 14;14(7):1539. doi: 10.3390/v14071539 (PMC9319728; doi:10.3390/v14071539)

The Cholesterol Transport Inhibitor U18666A Interferes with Pseudorabies Virus Infection

Byeongwoon Song[1, 2, *]

[1]Department of Microbiology, Immunology, and Physiology, Meharry Medical College, 1023 21st Avenue North, Nashville, TN 37208, USA

[2]Center for AIDS Health Disparities Research, Meharry Medical College, 1023 21st Avenue North, Nashville, TN 37208, USA

*Correspondence: Department of Microbiology, Immunology, and Physiology, Meharry Medical College, 1023 21st Avenue North, Nashville, TN 37208, USA; bsong@mmc.edu; Tel. (615) 327-6698; Fax. (615) 327-6021

Figure S1. A full image of WES protein analysis.

The cropped areas presented in Figure 5 are highlighted in red.

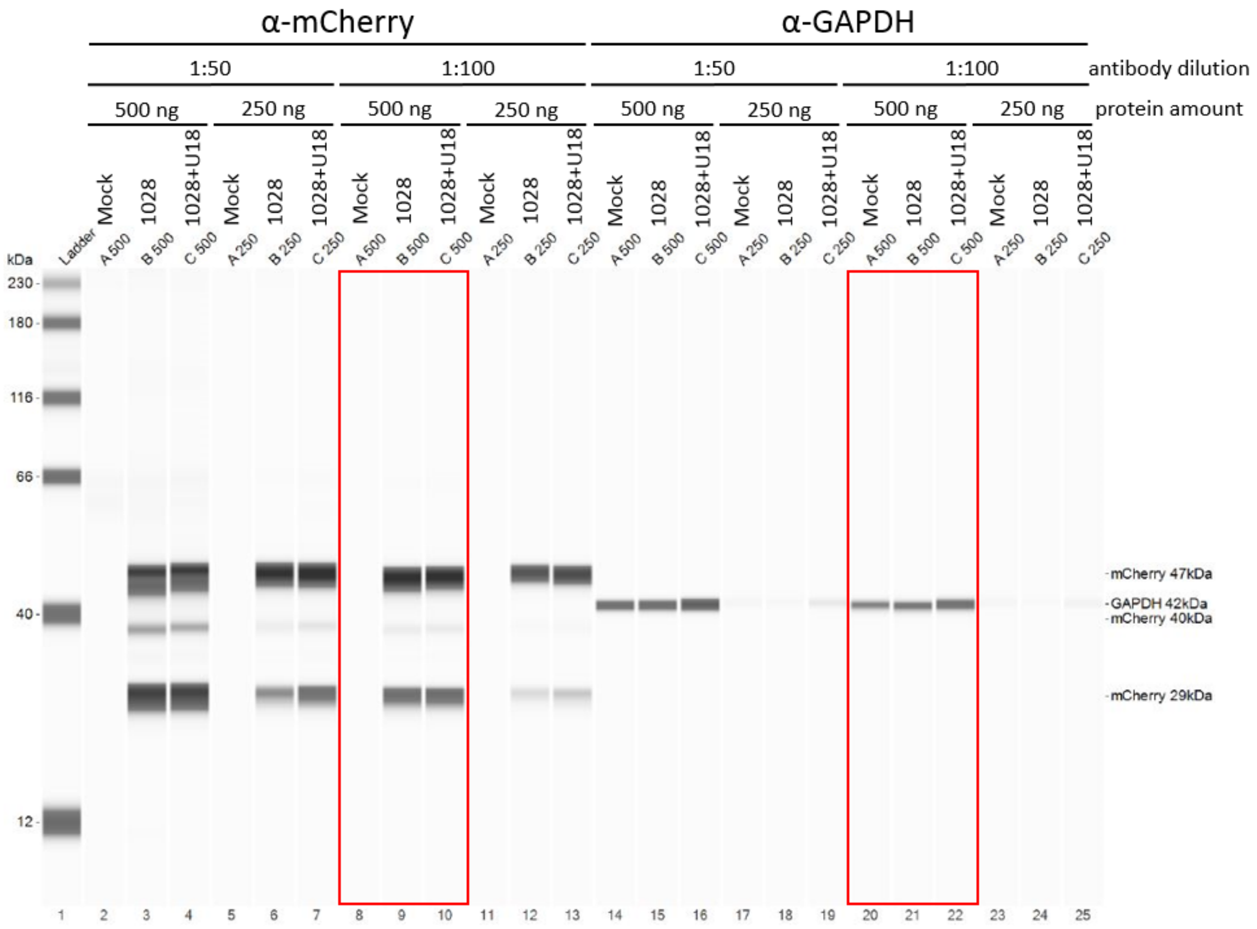

Supplement: Supplementary file 1 [file viruses-14-01539-s001.zip › viruses-1815681-supplementary.pdf]
